# Association between social support and medication literacy in older adults with hypertension

**DOI:** 10.3389/fpubh.2022.987526

**Published:** 2022-11-07

**Authors:** Zhiying Shen, Siqing Ding, Shuangjiao Shi, Zhuqing Zhong

**Affiliations:** ^1^Department of Hematology, Third Xiangya Hospital, Central South University, Changsha, China; ^2^Clinical Nursing Safety Management Research Center of Central South University, Third Xiangya Hospital, Central South University, Changsha, China; ^3^Department of Nursing, Third Xiangya Hospital, Central South University, Changsha, China

**Keywords:** hypertension, medication literacy, social support, relationship, older adult

## Abstract

**Background:**

Reduced physical function and reduced social networks place older adults with hypertension at high risk for medication-related harm. Medication literacy is one of the preventable factors that affect the success of drug therapy for hypertension. However, little is known about the level of medication literacy and its influencing factors in older adults with hypertension.

**Objective:**

The purpose of this study was to investigate the levels of social support and medication literacy, and the association between them in older Chinese adult patients with hypertension.

**Methods:**

A total of 362 older adult patients with hypertension were investigated using a demographic characteristics questionnaire, the Chinese Medication Literacy Scale for Hypertensive Patients (C-MLSHP) and the Social Support Rating Scale (SSRS). Pearson correlation analysis, canonical correlation analysis (CCA) and hierarchical linear regression were used to analyse the relationship between social support and medication literacy.

**Results:**

Our results showed that the mean scores for the C-MLSHP and the SSRS for older adult patients with hypertension were 23.89 (SD = 4.66) and 39.22 (SD = 5.53), respectively. The results of the Pearson correlation analysis suggested that the score for social support was positively correlated with the score for medication literacy (*r* = 0.431, *P* < 0.01). The results of CCA demonstrate that older adult patients with hypertension who had more subjective (*r*_s_ = 0.682) and objective support (*r*_s_ = 0.817) performed better in knowledge (*r*_s_ = 0.633), skills (*r*_s_ = 0.631) and behavior literacy (*r*_s_ = 0.715). Hierarchical linear regression indicated that two dimensions of subjective support (*B* = 0.252, *P* < 0.001) and objective support (*B* = 0.690, *P* < 0.001) in social support were found to be independent predictors of medication literacy (*R^2^* = 0.335*, F* = 19.745, *P* < 0.001).

**Conclusion:**

Social support is positively associated with medication literacy in older Chinese adult patients with hypertension. The study highlights the importance of social support in promoting medication literacy among older adult patients with hypertension.

## Introduction

Hypertension is a common disease characterized by elevated arterial pressure, and it is a major risk factor for many common causes of morbidity, including stroke, myocardial infarction, congestive heart failure, and end-stage renal disease (1). In 2019, the total number of people with hypertension was estimated to exceed 1.2 billion globally (2). As the most populous developing country, China faces a serious burden of hypertension. According to the data published in the “China Cardiovascular Disease Report 2021,” approximately 18.8% of Chinese adults (aged ≥ 18 years) were diagnosed with hypertension, and almost 50% of them were older adults (aged ≥ 60 years) (3).

Antihypertension medication still serves as a major therapy choice for treating hypertension. Most hypertensive patients need to take antihypertensive medications consistently and correctly to maintain blood pressure within target levels and to reduce the risk of complications and death. Medical therapy, however, is a double-edged sword. Medication-related harm (MRH) caused by non-adherence, medication error and improper medication use are major hidden dangers that threaten patients' health and waste medical resources. Studies have revealed that older adults with chronic conditions are high-risk groups for the occurrence of MRH due to their high exposure to medicines, difficulty in monitoring prescribed medications, and age-related pharmacokinetic and pharmacodynamic changes (4–7). Unsafe medication practices are very common in older adults, which results in a variety of adverse consequences. A systematic review found that MRH was the reason for hospitalization in one in ten hospitalized older adults (8), and another study estimated that avoiding hospitalization of one older people due to MRH could save approximately 5,600 euros (9). In a prospective study analyzing the risk of MRH in older hospitalized patients, 12% of patients experienced MRH events associated with antihypertensive medications. Eighty percent of MRH events were classified as “severe,” requiring dose changes or discontinuation of treatment. Moreover, further analysis showed that almost half of MRH cases could be prevented (10). The degradation of physiological and cognitive functions caused by aging, such as the reduction of vision, attention and memory and the decline of executive ability and judgment, all interfere with safe medication practice in older adults (11–13). On the other hand, the lack of pharmaceutical guidance services and the complexity of medication-related information (prescriptions and drug instructions) also increase the difficulty of obtaining, comprehending, communicating, calculating and processing medication-related information (14–16). Therefore, the problem of safe medication use in older adult patients with hypertension is worthy of attention.

The level of medication-related health literacy affects whether patients with chronic diseases can safely adhere to medication in the long term. Medication literacy is a new concept in the field of medicine that was proposed in recent years; it is defined as “the degree to which individuals can obtain, comprehend, communicate, calculate and process patient-specific information about their medications to make informed medication and health decisions in order to safely and effectively use their medications, regardless of the mode by which the content is delivered (e.g., written, oral and visual)” (17, 18). Medication literacy reflects the specific literacy competencies required to act on medication-related information (19). Our research team has conducted a series of exploratory studies on the medication literacy of hypertensive patients, which showed that knowledge, attitude, skills and behavior are the four core elements of hypertensive patients' medication literacy, and each domain is essential and critical for processing medication information and correct medication use (20–23). First, correct and adequate knowledge about hypertension and its medication treatment is fundamental to safe medication use and persistent adherence (24, 25). Second, hypertensive patients' attitudes, including their beliefs related to hypertension severity and susceptibility, their beliefs about the effectiveness or necessity of taking antihypertensive medication, and their self-efficacy in hypertension disease management, also play critical roles in their adherence to antihypertensive drug use and disease management (26, 27). Third, appropriate skills to calculate and process information about their medications are prerequisites for making informed medication and health decisions to achieve safe and e?ective medication behavior (25, 28). Finally, correct medication use is one of the indicators of optimal goals and outcomes in the process of medication use (17, 20). Specifically, patients' medication-taking behavior, decision-making regarding type of medication, medication information-seeking, blood pressure monitoring, and responses to adverse effects.

Previous studies have found that medication literacy is closely related to appropriate medication use and safe self-care (23, 29). The improvement of medication literacy could help reduce non-adherence to treatments, enabling patients to participate more fully in their medication therapy (23, 30). In recent years, some studies have investigated the medication literacy level of hypertensive patients and found that the medication knowledge level among the older adult group was lower than that of other age groups (19, 21, 22). The prevalence of inadequate levels of literacy is high among older adults, making them more susceptible to medication errors (31). These studies, however, did not specifically focus on older adult patients with hypertension and did not explore the relationship between social support and medication literacy.

Social support was defined as “the social resources that individuals perceive to be available or that are actually provided to them by non-professionals in the context of both formal support groups and informal helping relationships” (32). Xiao (33) further categorized social support into three dimensions: subjective support, objective support, and utilization of support. Social support can be provided by family members, close friends, colleagues, neighbors, relatives, and healthcare personnel. Subjective support is the emotional and spiritual support obtained by individuals, which can result in psychological satisfaction and a sense of being respected. Objective support refers to the actual help and the visible assistance given by organizations and social relations in which the individual is located. The utilization of support indicates that patients are seeking social support and their degree of acceptance of social support.

Prior studies have demonstrated that receiving social support from formal or informal social networks can help hypertensive patients improve their medication adherence and self-care behaviors and even reduce the risk of hypertension (34–36). In addition, studies have shown that inadequate social support may be a direct or indirect predictor of poor health literacy among patients with hypertension (37, 38). Therefore, we speculate that medication literacy, as in health literacy with regard to the process of medication use, may be associated with social support. In fact, in the conceptual model of medication literacy proposed by Neiva et al. (19), medication literacy can be considered an interaction between the demands of the social environment, including the health system, and the competencies of individuals to use the medication properly; thus, the supportiveness of family and social networks are emerging as important social and environmental determinants of medication literacy. This theory has been supported by a study that revealed a significant association of social support with medication literacy in Chinese patients with coronary heart disease (39). However, there is a lack of empirical evidence exploring the relationship between medication literacy and social support in older adult patients with hypertension. Assessing the medication literacy of older adults with hypertension can provide insight into their ability to acquire, understand, process, and apply medication-related information. Meanwhile, analyzing the impact of social support on medication literacy can provide a valuable reference for formulating strategies to improve the medication literacy of older adult patients with hypertension, improving safe medication practice and preventing MRH.

To our knowledge, this study is the first to investigate the level of medication literacy and explore the relationship between social support and medication literacy in older adult patients with hypertension. Meanwhile, based on the extant literature and related theory of medication literacy, we hypothesized that social support is associated with medication literacy in older adult patients with hypertension.

## Methods

### Study design and participants

This cross-sectional study was conducted at three general hospitals and three community healthcare services in a southern province of China from June 2020 to January 2021. Patients were included if they (1) were aged 60 years or older; (2) had been diagnosed with hypertension by a cardiologist; (3) were being treated with antihypertensive medication; (4) spoke Chinese and communicated well with others; and (5) had an understanding of the purpose and process of the study and gave informed consent. Patients were excluded if they (1) were diagnosed with other serious diseases, such as other cancers, acute myocardial infarction, cerebral hemorrhage or chronic renal failure; (2) had secondary hypertension, such as elevated blood pressure caused by chronic renal dysfunction diseases; or (3) were diagnosed with psychological or mental impairment according to International Classification of Diseases guidelines or were receiving psychotherapy treatment. Investigations were conducted by two trained researchers. The diagnosis of hypertension and the number of antihypertensive medications that patients were taking were abstracted from medical records. Hypertensive patients who were eligible were invited to participate in the study while they were waiting for clinical consultations or after the clinic visit in the waiting room of the outpatient clinic. Each participant was provided with information on the purpose, content, investigation procedures and anonymity of respondents. Upon signing an informed consent form, each patient completed a self-administered (self-completed) questionnaire. For illiterate patients, the researchers communicated with both them and their family members. If they agreed to participate in the study, then they were instructed by one of their family members to sign the informed consent forms. Next, the researchers read the items word by word, and the responses of the subjects were recorded on the questionnaires. All completed questionnaires were immediately collected onsite and checked for missing information to ensure data integrity.

### Measures

#### Sociodemographic and clinical characteristics

The following information about patients' sociodemographic and clinical characteristics was collected using a homemade questionnaire: gender, education level, annual income, marital status, duration of hypertension, family history of hypertension, number of antihypertensive drugs prescribed, and number of cohabitating persons.

#### Chinese medication literacy scale for hypertensive patients

The C-MLSHP is a self-administered medication literacy measure for hypertensive patients (20). This scale includes 37 items on four domains of knowledge literacy (e.g., “Hypertension can be induced by weight gain and obesity.”), attitude literacy (e.g., “Patients with hypertension should visit doctors periodically.”), skill literacy (e.g., “According to this prescription sheet, how many times a day should Tom take antihypertensives in total?”), and behavior literacy (e.g., “Have you ever searched for any information about antihypertensives?”). The scale-level content validity index (S-CVI/Ave) of this scale was 0.97, and the I-CVI for each item ranged from 0.83 to 1.00, indicating a good and acceptable content and face validity. Cronbach's α coefficient was 0.85 for the full scale, 0.75 for the knowledge dimension, 0.78 for the attitude dimension, 0.76 for the skill dimension, and 0.74 for the behavior dimension. Spearman-Brown split-half reliability was 0.89 for the full scale, 0.82 for the knowledge dimension, 0.87 for the attitude dimension, 0.79 for the skill dimension, and 0.81 for the behavior dimension. The test–retest reliability was 0.97 for the full scale, 0.96 for the knowledge dimension, 0.96 for the attitude dimension, 0.88 for the skill dimension, and 0.93 for the behavior dimension (20). For items in the domains of knowledge and skill, a correct answer for each item scored 1, and an incorrect or “I don't know” response scored 0. Each item in the attitude domain was measured on a 5-point Likert scale: 1.00 = Totally Agree, 0.75 = Agree, 0.5 = Do Not Agree, 0.25 = Disagree, 0.00 = Totally Disagree. In the practice domain, responses were also measured on a 5-point Likert scale: always, often, sometimes, seldom, never), for scores of 1.0, 0.75, 0.5, and 0.25, respectively. The total score for this scale ranged from 0 to 37, with higher scores indicating higher medication literacy levels. The scale has been attached as Appendices in the Supplementary material.

#### Social support rating scale

This scale is used to measure individual social relations. It includes 10 items in three dimensions: subjective support (e.g., “How many intimate friends do you have? From whom can you receive support and help?”), objective support (e.g., “In the past, when you encountered difficulties, what was your source of comfort and caring?”), and utilization of social support (e.g., “What is the way of talking when you are in trouble?”) (33). The highest possible score is 66, with higher scores indicating better social support. Scores are categorized as follows: scores < 22 indicate a low level, scores 23~44 indicate a medium level, and scores 45~66 indicate a high level. The Cronbach's α coefficient was 0.81 for the full scale, 0.72 for the subjective support dimension, 0.73 for the objective support dimension, and 0.81 for the utilization of support dimension. The scale and scoring method are shown in Supplementary material.

### Data analysis

Data were analyzed using IBM SPSS Statistics (Version 24.0, Armonk, NY, USA). Continuous variables are expressed as the means ± standard deviations (M ± SD), and categorical variables are summarized as absolute numbers and percentages. The scores of medication literacy among participants with different sociodemographic and clinical characteristics were compared using the independent-sample *t* test or analysis of variance with the LSD *post-hoc* test. Three strategies were employed to determine the association between medication literacy and social support. First, Pearson correlation analysis was used to determine the correlation between medication literacy and social support. Second, a canonical correlation analysis (CCA) was conducted using the three social support variables (X_1_: Subjective support; X_2_: Objective support; X_3_: Support utilization) as predictors of the four medication literacy variables (Y_1_: Knowledge literacy; Y_2_: Attitude literacy; Y_3_: Skill literacy; Y_4_: Behavior literacy) to further examine the relationships between the multiple dimensions of medication literacy and social support. Canonical variables U and V represent the comprehensive variables formed by X_1_-X_3_ and Y_1_-Y_4_, respectively. CCA is a method of identifying associations between two multidimensional variables, which has the advantage of being able to examine multiple causes and multiple effects between complex constructs and to identify a variable's importance in terms of degree (40). Third, hierarchical linear regression analysis was conducted with the scores of medication literacy as dependent variables. The clinical and socio-demographic variables demonstrated statistical significance at the level of *P* < 0.05 in the univariate analysis were entered into the model as independent variables on the first step (including gender, education level, annual income, duration of hypertension, number of antihypertensive drugs prescribed and number of co-lived persons), and the scores of each dimension of social support on the second step. Hierarchical linear regression analysis was performed according to the standard of α _in_ = 0.05 and α _out_ = 0.10 with the Enter method. Two-sided *P*-values < 0.05 were considered statistically significant.

## Results

### Patient characteristics

In this study, a total of 400 questionnaires were distributed, of which 387 were completed, yielding a response rate of 96.75%. Of these, there were 362 valid questionnaires, for a valid rate of 90.5%. The demographic and clinical characteristics of the 362 participants in the study are presented in Table 1. The mean age of the participants was 67.3 years (SD = 6.7 years). Approximately half of the patients (51.7%) were male, 60% had a lower level of education (in China, every citizen must complete 6 years of primary school education and 3 years of junior middle school education), and 30.4% had a family annual income of 30,000–49,999 Chinese yuan. The majority of the patients in the study population were married (88.4%). A total of 37.8% of the participants had been diagnosed with hypertension for at least 10 years, 54.1% had a family history of hypertension, 64.4% were taking only one antihypertensive drug, and only 8.8% lived alone or with one person.

**Table 1:** Patient characteristics (*n* = 362).

**Factors**	**Items**	***N***	**%**	**Medication literacy**	***t/F* value**	***P-*value**
Gender	Male	187	51.7	24.35 ± 4.98	1.986	0.048
	Female	175	48.3	23.39 ± 4.24		
Education level	Junior middle school or below	217	60	22.56 ± 4.73	30.746	0.000
	Senior high school or secondaryspecialized school	88	24.3	24.94 ± 3.37^a^		
	Junior college or above	57	15.7	27.30 ± 3.93^ab^		
Annual income	< 30,000/year	150	41.4	22.97 ± 4.91	5.419	0.005
	30,000–49,999/year	110	30.4	24.80 ± 4.38^a^		
	≥50,000/year	102	28.2	24.25 ± 4.36^a^		
Marital status	Married	320	88.4	23.93 ± 4.71	0.032	0.974
	Single	42	11.6	23.54 ± 4.27		
Duration of hypertension	< 5 years	106	29.3	22.97 ± 4.89	7.153	0.001
	5–9.9 years	119	32.9	25.15 ± 4.59^a^		
	≥10 years	137	37.8	23.50 ± 4.31^b^		
Family history of hypertension	Yes	196	54.1	24.28 ± 4.45	1.746	0.082
	No	166	45.9	23.42 ± 4.86		
Number of antihypertensive drugs prescribed	One	233	64.4	23.57 ± 4.07	5.639	0.004
	2–3 kinds	118	32.6	24.81 ± 5.29^a^		
	4 or more	11	3	20.66 ± 6.81^ab^		
Hypertension complication	Yes	121	33.4	23.28 ± 4.93	1.739	0.083
	No	241	66.6	24.19 ± 4.49		
Number of co-lived person	1 or 0	32	8.8	23.30 ± 4.34	10.587	0.000
	2–4	194	53.6	24.89 ± 4.84		
	≥5–7	136	37.6	22.59 ± 4.11^b^		

a Compared with the first layer, *P* < 0.05;

b Compared with the second layer, *P* < 0.05.

### Medication literacy of older adults with hypertension

The mean score for medication literacy was 23.89 ± 4.66 (range: 11.50–35.00). Additionally, the mean scores for each dimension of medication literacy were 6.35 ± 2.15 (range: 1.00–9.00) for the knowledge dimension, 5.18 ± 1.08 (range: 2.75–7.75) for the attitude dimension, 4.33 ± 2.15 (range: 0.00–7.00) for the skill dimension, and 8.03 ± 2.01 (range: 3.00–12.00) for the behavior dimension. Patients with different sociodemographic and clinical characteristics had different medication literacy scores, and the results of independent-sample *t* tests and analysis of variance for each item are shown in Table 1.

### Social support for older adults with hypertension

The results showed that the mean score of social support was 39.22 ± 5.53 (range: 28.00–53.00), and the mean scores for subjective support, objective support and utilization of support were 22.87 ± 4.23 (range: 14.00–31.00), 9.04 ± 2.22 (range: 1.00–16.00), and 7.31 ± 1.95 (range: 3.00–12.00), respectively. According to the scoring standard of the SSRS, the score for social support of older adult patients with hypertension was at a medium level. Specifically, 32 (8.8%) had low social support, 265 (73.2%) had medium social support, and 65 (18.0%) had high social support.

### Association between medication literacy and social support

First, the results of the Pearson correlation analysis suggested that the total scores of social support (*r* = 0.431, *P* < 0.01), subjective support (*r* = 0.316, *P* < 0.01) and objective support (*r* = 0.408, *P* < 0.01) were positively correlated with the total score of medication literacy, respectively. However, the utilization support dimension was found to be positively correlated only with behavior literacy (*r* = 0.182, *P* < 0.01) (Table 2).

**Table 2:** Pearson correlation analysis between medication literacy and social support (*n* = 362).

**Dimensions**	**Subjective support**	**Objective support**	**Utilization of support**	**Total social support scores**
Knowledge literacy	0.164^**^	0.325^**^	0.041	0.242^**^
Attitude literacy	0.106^*^	0.221^**^	0.043	0.155^**^
Skill literacy	0.241^**^	0.236^**^	0.046	0.296^**^
Behavior literacy	0.244^**^	0.234^**^	0.182^**^	0.345^**^
Total medication literacy scores	0.316^**^	0.408^**^	0.072	0.431^**^

* *P* < 0.05;

** *P* < 0.01.

Second, the results of the CCA are shown in Table 3. Two pairs of canonical variables (U_1_V_1_, U_2_V_2_) were generated with canonical correlation coefficients of 0.484 (Wilks's λ = 0.727, *F* = 10.031, *P* < 0.001) and 0.218 (Wilks's λ = 0.949, *F* = 3.157, *P* = 0.005). The standardized canonical correlation coefficient and canonical loadings of U_1_V_1_ and U_2_V_2_ are presented in Table 3. Redundancy analysis performed on the two pairs of canonical variables demonstrated that U_1_V_1_ and U_2_V_2_ explained 39.4 and 30.2% of the variance of the observed social support variables and 37.1 and 12.3% of the variance of the observed medication literacy variables, respectively. Since U_1_V_1_ had the largest canonical correlation coefficient and the highest contribution rate, which could fully express the correlation between the two groups of variables, U_1_V_1_ was selected for interpretation in this study. The normalized expression for U_1_V_1_ is as follows:


$$\begin{array}{l} U_{1}=0.495X_{1}+0.3729X_{2}+0.300X_{3}\\ V_{1}=0.344Y_{1}+0.163Y_{2}+0.466Y_{3}+0.587Y_{4} \end{array}$$

**Figure 1:**
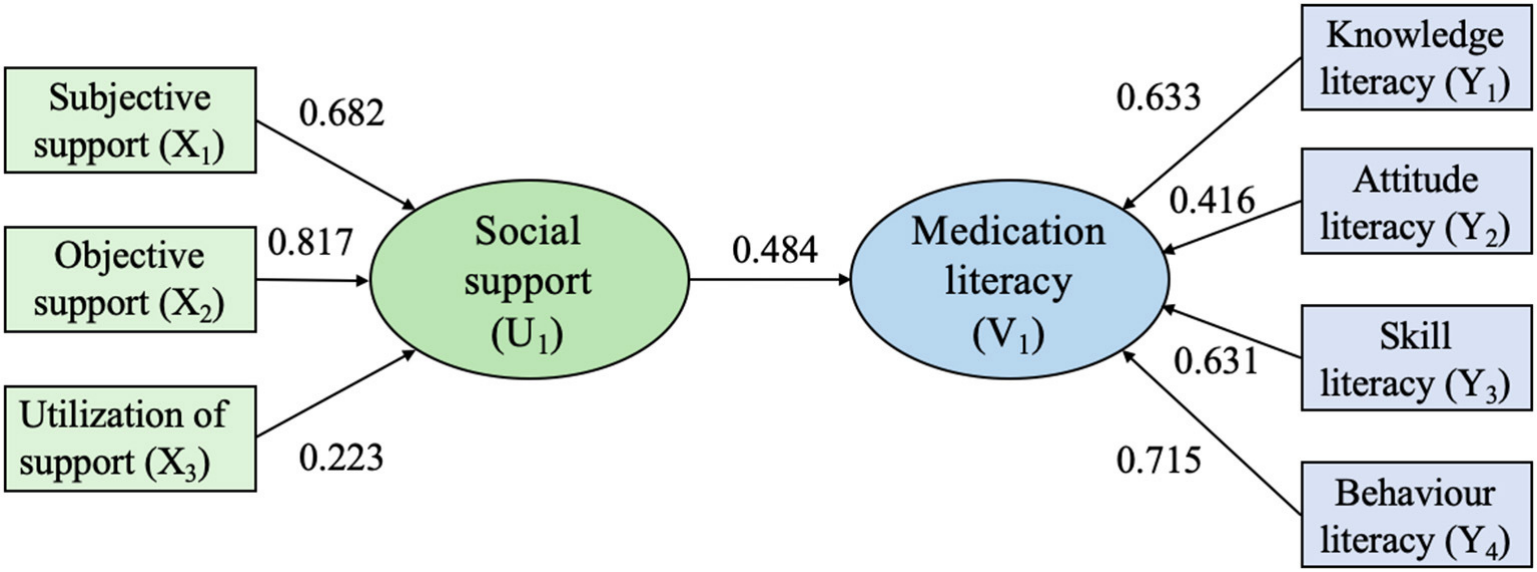
Conceptual model for the canonical correlation.

From the expression, it is clear that U_1_ was mainly affected by subjective support (X_1_) and objective support (X_2_), and V_1_ had a large weight in knowledge literacy (Y_1_), skill literacy (Y_3_) and behavior literacy (Y_4_). Because all of the observed variables had the same sign, the results indicated that older adult patients with hypertension with higher subjective and objective support performed better in the three dimensions of knowledge literacy, skill literacy and behavioral literacy (Figure 1).

**Table 3:** Canonical correlation analysis of medication literacy and social support.

**Variable**	**U**_**1**_ **V**_**1**_		**U**_**2**_ **V**_**2**_	
	**Coef**	***r_***s***_***	**Coef**	***r_***s***_***
Subjective support (X_1_)	0.495	0.682	0.377	0.261
Objective support (X_2_)	0.729	0.817	0.522	0.519
Support utilization (X_3_)	0.300	0.223	0.760	0.830
Knowledge literacy (Y_1_)	0.344	0.633	0.688	0.631
Attitude literacy (Y_2_)	0.163	0.416	0.448	0.496
Skill literacy (Y_3_)	0.466	0.631	0.207	0.012
Behavior literacy (Y_4_)	0.587	0.715	0.686	0.498

Coef, standardized canonical correlation coefficient; r_*s*_, canonical loadings.

Finally, the results of hierarchical linear regression analysis showed that independent variables such as gender explained 17.7% of the variance of medication literacy in the first layer (*R^2^* = 0.177*, F* = 12.695, *P* < 0.001). Social support variables were added on the basis of Model 1, and the power to explain the variance of medication literacy increased by 15.8% in Model 2 (*R^2^* = 0.335*, F* = 19.745, *P* < 0.001). The final hierarchical regression model explained 33.5% of variability in medication literacy. Education level (*B* = 1.068, *P* = 0.001), annual income (*B* = 0.741, *P* = 0.007), number of co-lived person (*B* = −1.521, *P* < 0.001), subjective support (*B* = 0.252, *P* < 0.001) and objective support (*B* = 0.690, *P* < 0.001) were the factors affecting patients' medication literacy (Table 4).

**Table 4:** Hierarchical linear regression analysis of factors influencing medication literacy (*n* = 362).

**Variables**	**Model 1**				**Model 2**			
	**B**	**SE**	***t***	***P***	**B**	**SE**	***t***	***P***
Constant	22.503	1.629	13.814	< 0.001	10.741	2.095	5.126	< 0.001
Gender	−0.777	0.502	−1.547	0.123	−0.319	0.458	−0.696	0.487
Education level	2.210	0.311	7.106	0.000	1.068	0.307	3.476	**0.001**
Annual income	0.201	0.295	0.682	0.496	0.741	0.274	2.704	**0.007**
Duration of hypertension	0.227	0.303	0.750	0.454	−0.196	0.281	−0.698	0.485
Number of antihypertensive drugs prescribed	0.492	0.490	1.005	0.315	0.867	0.444	1.954	0.052
Number of co-lived person	−1.060	0.373	−2.841	0.005	−1.521	0.342	−4.441	<**0.001**
Subjective support					0.252	0.052	4.867	<**0.001**
Objective support					0.690	0.100	6.914	<**0.001**
Support utility					0.175	0.107	1.635	0.103
*R^2^*	0.177				0.335			
*F* value	*F*_(6, 355)_ = 12.695, *P* < 0.001				*F*_(9, 352)_ = 19.745, *P* < 0.001			

B, unstandardized regression coefficients; SE, Standard error.

## Discussion

This study is the first to describe the association between social support and medication literacy among older Chinese adult patients with hypertension. In this study, we found that social support had a positive relationship with medication literacy, and older adult patients with hypertension who had more subjective and objective support performed better in knowledge, skills and behavior literacy. Furthermore, two dimensions of subjective support and objective support in social support were found to be independent predictors of medication literacy.

### Medication literacy of older adult patients with hypertension

Our results showed that the mean score for the C-MLSHP for older adult patients with hypertension was 23.89 ± 4.66, which is lower than the survey results of hypertensive patients in the 18–44 years (27.10 ± 4.77) and 45–59 years (24.87 ± 5.02) age groups by Ma et al. (22). Although some studies used different medication literacy assessment tools than this study, their results also showed that medication literacy decreased with increasing patient age (41, 42). The results showed that education level, annual income and number of cohabitating persons were the demographic factors affecting the level of medication literacy among older adult patients with hypertension. Similar influencing factors of medication literacy for hypertensive patients have also been identified in previous studies (20, 22). Patients with higher education level have better ability to obtain, understand and calculate information related to complex medication, and are more able to make correct medication decisions (43). Therefore, they have higher medication literacy than patients with lower education level. In addition, the results in the present study showed that with the increase of annual income, the level of medication literacy of the patients will be improved. One possible reason is that patients with higher annual incomes are more likely to value the health education of medical staff than those with lower annual incomes. Secondly, patients with higher annual incomes are more willing to spend money on health care and health promotion than patients who lack sufficient and stable sources of income, including long-term purchase and use of antihypertensive drugs (22, 44). A study showed that a lack of family support caused by ≤ 2 people living together is one of the main risk factors for hypertension in men (45). However, this study found that the medication literacy of older adult patients with hypertension is reduced when the number of cohabitating persons is more than five. The reason might be that living with more family members does not mean that patients can obtain more effective family support. In contrast, they may be distracted from their health by their families.

### Social support of older adult patients with hypertension

In this study, the social support score was 39.22 ± 5.53, which revealed that older adult patients with hypertension received mid-level social support. Furthermore, the scores of objective support, subjective support and support utilization were lower than the norm level of Chinese adults' social support (44.34 ± 8.38) (46). The reason for this phenomenon may be that the older population in China have fewer social activities after retirement or are unable to work. Their sources of social support are mainly family members, such as spouses and children, while support from other social networks, such as friends, organizations and professional medical personnel, is limited. A qualitative study found that with increasing age, the demand for social support of patients with hypertension also gradually increases (47). Therefore, if family members of older hypertensive people lack time and energy to care for them, their social support level, including subjective support and objective support, will be further reduced (48).

### Relationship between social support and medication literacy

The results of the present study revealed the important impact of social support on medication literacy in older adult patients with hypertension. When taken as a whole, the results of Pearson correlation analysis suggested that social support was positively correlated with medication literacy (*r* = 0.431, *P* < 0.01). This result was consistent with a previous study in which the impact of social support on medication literacy among patients with coronary heart disease was tested (39). It indicates that strengthening patient social support can, to some extent, improve patient medication literacy levels.

To further analyse how social support influences medication literacy, CCA was used. According to the first pair of canonical correlation variables (U_1_V_1_), the comprehensive variables reflecting social support (U_1_) are mainly determined by objective support and subjective support, and the comprehensive variables reflecting medication literacy are mainly determined by behavior literacy, knowledge literacy and skill literacy. Therefore, the results suggest that older adult patients with hypertension with more subjective and objective support perform better in knowledge, skills and behavior literacy.

Moreover, the results of hierarchical regression analysis showed that subjective support (*B* = 0.252, *P* < 0.001) and subjective support (*B* = 0.175, *P* < 0.001) had a significant impact on medication literacy level in older adult with hypertension. Subjective support and objective support independently explained 15.8% of the variation of medication literacy, suggesting that the increase of subjective support and objective support would possibly improve medication literacy of older adult patients with hypertension. This result was consistent with a previous study, in which the relationship between social support and medication literacy among discharged patients after percutaneous coronary intervention was tested (49). These findings suggest the potential value of social support in promoting medication literacy among older adult patients with hypertension. Strengthening subjective support and objective support for older adult patients with hypertension may help to improve their medication literacy.

However, in the CCA and regression analysis, no relationship was found between the dimension of support utilization and medication literacy. This was consistent with the study of Qiao et al. (39). The study reported by Lu and Chen (50) indicated that with increasing age, the physiological function of individuals degenerates, resulting in inconvenient movement, cognitive function degradation and slow behavioral responses. Therefore, these negative changes prevent them from actively using social support to a certain extent. Considering this contradiction, further studies might be required to explain the interaction between support utilization and medication literacy.

## Study limitations

This study had some limitations. First, we only selected older adult patients with hypertension in Changsha, Hunan Province, China, which resulted in a small sample size. Second, this study adopted an across-sectional design and lacked follow-up of survivors across different time periods. Further studies using a multicentre, large-sample longitudinal cohort are needed to validate the observed trajectory of medication literacy, examine its dynamic changes, and provide a reference for the clinical development of targeted interventions.

## Conclusion

This study was conducted to examine the relationship between medication literacy and social support among older adults Chinese patients with hypertension. Higher social support was found to be associated with better medication literacy. In the absence of other studies on older adult patients with hypertension, our study provides important information for medical health providers to conduct medication literacy interventions for older adult patients with hypertension. Further prospective studies and intervention trials are warranted.

## Data availability statement

The raw data supporting the conclusions of this article will be made available by the authors, without undue reservation.

## Ethics statement

The studies involving human participants were reviewed and approved by Ethics Committee of the Third Xiangya Hospital. The patients/participants provided their written informed consent to participate in this study.

## Author contributions

ZS performed the statistical analysis and drafted the original manuscript. ZZ and SS designed the instrument, collected the data, and participated in revision of the paper. SD conceptualized the study and controlled the quality of this study. All authors read and approved the final manuscript.

## Funding

This research was supported by the Hunan Clinical Medical Technology Innovation and Guidance Project (Project Number: 2021SK53705).

## Conflict of interest

The authors declare that the research was conducted in the absence of any commercial or financial relationships that could be construed as a potential conflict of interest.

## Publisher's note

All claims expressed in this article are solely those of the authors and do not necessarily represent those of their affiliated organizations, or those of the publisher, the editors and the reviewers. Any product that may be evaluated in this article, or claim that may be made by its manufacturer, is not guaranteed or endorsed by the publisher.

## References

[1] World Health Organization. A Global Brief on Hypertension. Available online at:http://apps.who.int/iris/bitstream/10665/79059/1/WHO_DCO_WHD_2013.2_eng.pdf (accessed May 20, 2022).

[2] NCD Risk Factor Collaboration. Worldwide trends in hypertension prevalence and progress in treatment and control from 1990 to 2019: a pooled analysis of 1201 population-representative studies with 104 million participants. Lancet. (2021) 398:957–80. 10.1016/S0140-6736(21)01330-1 34450083 PMC8446938

[3] The Writing Committee of the Report on Cardiovascular Health and Diseases in China. Report on cardiovascular health and diseases burden in China: an Updated Summary of 2021. Chin J Cardiovasc Res. (2022) 20:577–96. 10.3969/j.issn.1672-5301.2022.07.001

[4] Kaufmann CP Stämpfli D Hersberger KE Lampert ML. Determination of risk factors for drug-related problems: a multidisciplinary triangulation process. BMJ Open. (2015) 5:e006376. 10.1136/bmjopen-2014-006376 25795686 PMC4368979

[5] Gurwitz JH Field TS Harrold LR Rothschild J Debellis K Seger AC. Incidence and preventability of adverse drug events among older persons in the ambulatory setting. JAMA. (2003) 289:1107–16. 10.1001/jama.289.9.1107 12622580

[6] Leendertse AJ Egberts AC Stoker LJ van den Bemt PM. Frequency of and risk factors for preventable medication-related hospital admissions in the Netherlands. Arch Intern Med. (2008) 168:1890–6. 10.1001/archinternmed.2008.3 18809816

[7] Hernandez SHD Cruz-Gonzalez I. Incidence and preventability of medication errors and ADEs in ambulatory care older patients. Consult Pharm. (2018) 33:454–66. 10.4140/TCP.n.2018.454 30068439

[8] Alhawassi TM Krass I Bajorek BV Pont LG A. systematic review of the prevalence and risk factors for adverse drug reactions in the elderly in the acute care setting. Clin Interv Aging. (2014) 9:2079–86. 10.2147/CIA.S71178 25489239 PMC4257024

[9] Leendertse AJ Van Den Bemt PM Poolman JB Stoker LJ Egberts AC Postma MJ. Preventable hospital admissions related to medication (HARM): cost analysis of the HARM study. Value Health. (2011) 14:34–40. 10.1016/j.jval.2010.10.024 21211484

[10] Hussain A Ali K Parekh N Stevenson JM Davies JG Bremner S. Characterising older adults' risk of harm from blood-pressure lowering medications: a sub-analysis from the PRIME study. Age Ageing. (2022) 51:afac045. 10.1093/ageing/afac045 35352796 PMC8966023

[11] Advinha AM Lopes MJ de Oliveira-Martins S. Assessment of the elderly's functional ability to manage their medication: a systematic literature review. Int J Clin Pharm. (2017) 39:1–15. 10.1007/s11096-016-0409-z 27942949

[12] Elliott RA Goeman D Beanland C Koch S. Ability of older people with dementia or cognitive impairment to manage medicine regimens: a narrative review. Curr Clin Pharmacol. (2015) 10:213–21. 10.2174/1574884710666150812141525 26265487 PMC5396255

[13] Leat SJ Krishnamoorthy A Carbonara A Gold D Rojas-Fernandez C. Improving the legibility of prescription medication labels for older adults and adults with visual impairment. Can Pharm J. (2016) 149:174–84. 10.1177/1715163516641432 27212968 PMC4860753

[14] Gentizon J Bovet E Rapp E Mabire C. Medication literacy in hospitalized older adults: concept development. Health Lit Res Pract. (2022) 6:e70–83. 10.3928/24748307-20220309-02 35389270 PMC8973764

[15] Maniaci MJ Heckman MG Dawson NL. Functional health literacy and understanding of medications at discharge. Mayo Clin Proc. (2008) 83:554–8. 10.4065/83.5.554 18452685

[16] Kripalani S Henderson LE Jacobson TA Vaccarino V. Medication use among inner-city patients after hospital discharge: patient-reported barriers and solutions. Mayo Clin Proc. (2008) 83:529–35. 10.4065/83.5.529 18452681

[17] Pouliot A Vaillancourt R Stacey D Suter P. Defining and identifying concepts of medication literacy: an international perspective. Res Social Adm Pharm. (2018) 14:797–804. 10.1016/j.sapharm.2017.11.005 29191647

[18] Raynor DK. Addressing medication literacy: a pharmacy practice priority. Int J Pharm Pract. (2010) 17:257–9. 10.1211/ijpp.17.05.0001 20214266

[19] Neiva Pantuzza LL Nascimento ED Crepalde-Ribeiro K Botelho SF Parreiras Martins MA Camila de Souza Groia Veloso R. Medication literacy: a conceptual model. Res Social Adm Pharm. (2022) 18:2675–82. 10.1016/j.sapharm.2021.06.003 34134939

[20] Zhong Z Shi S Duan Y Shen Z Zheng F Ding S. The development and psychometric assessment of chinese medication literacy scale for hypertensive patients (C-MLSHP). Front Pharmacol. (2020) 11:490. 10.3389/fphar.2020.00490 32425773 PMC7203424

[21] Zhong Z Ma G Zheng F Duan Y Ding S Luo A. Medication literacy in a cohort of Chinese patients discharged with essential hypertension. Front Public Health. (2019) 7:385. 10.3389/fpubh.2019.00385 31998676 PMC6962135

[22] Ma G Luo A Shen Z Duan Y Shi S Zhong Z. The status of medication literacy and associated factors of hypertensive patients in China: a cross-sectional study. Intern Emerg Med. (2020) 15:409–19. 10.1007/s11739-019-02187-0 31650433 PMC7165129

[23] Shi S Shen Z Duan Y Zhong Z. Association between medication literacy and medication adherence among patients with hypertension. Front Pharmacol. (2019) 10:822. 10.3389/fphar.2019.00822 31396088 PMC6664237

[24] Sauceda JA Loya AM Sias JJ Taylor T Wiebe JS Rivera JO. Medication literacy in Spanish and English: psychometric evaluation of a new assessment tool. J Am Pharm Assoc (2003). (2012) 52:e231–40. 10.1331/JAPhA.2012.11264 23229985

[25] King SR McCaffrey DJ Bouldin AS. Health literacy in the pharmacy setting: defining pharmacotherapy literacy. Pharm Pract. (2011) 9:213–20. 10.4321/S1886-36552011000400006 24198859 PMC3818737

[26] Al-Noumani H Wu JR Barksdale D Sherwood G AlKhasawneh E Knafl G. Health beliefs and medication adherence in patients with hypertension: a systematic review of quantitative studies. Patient Educ Couns. (2019) 102:1045–56. 10.1016/j.pec.2019.02.022 30846205

[27] Qvarnström M Kahan T Kieler H Brandt L Hasselström J Wettermark B. Medication persistence to antihypertensive drug treatment - a cross-sectional study of attitudes towards hypertension and medication in persistent and non-persistent patients. Blood Press. (2019) 28:309–16. 10.1080/08037051.2019.1627858 31203660

[28] Horvat N Vidic L Kos M. Development and content validation of medication literacy assessment questionnaire. Int J Clin Pharm. (2017) 39:606. 10.1007/s11096-017-0462-2

[29] Shen Z Ding S Zheng F Duan Y Shi S Zhong Z. Development and implementation of a medication literacy promotion program for patients with hypertension. J Nurs Sci. (2019) 34:87–91. 10.3870/j.issn.1001-4152.2019.10.087

[30] Chang FC Chi HY Huang LJ Lee CH Yang JL Yeh MK. Developing school-pharmacist partnerships to enhance correct medication use and pain medication literacy in Taiwan. J Am Pharm Assoc. (2015) 55:595–602. 10.1331/JAPhA.2015.15053 26409206

[31] Cutilli CC. Health literacy in geriatric patients: an integrative review of the literature. Orthop Nurs. (2007) 26:43–8. 10.1097/00006416-200701000-00014 17273109

[32] Cohen S Gottlieb BH Underwood LG. Social relationships and health: challenges for measurement and intervention. Adv Mind Body Med. (2001) 17:129–41. 11335207

[33] Xiao S. The theoretical basis and research application of “Social Support Rating Scale”. J Clin Psychiatry. (1994) 4:98–100.

[34] Johnson VR Jacobson KL Gazmararian JA Blake SC. Does social support help limited-literacy patients with medication adherence? a mixed methods study of patients in the pharmacy intervention for limited literacy (PILL) study. Patient Educ Couns. (2010) 79:14–24. 10.1016/j.pec.2009.07.002 19647967

[35] Harding BN Hawley CN Kalinowski J Sims M Muntner P Mielcarek BAY. Relationship between social support and incident hypertension in the Jackson heart study: a cohort study. BMJ Open. (2022) 12:e054812. 10.1136/bmjopen-2021-054812 35301208 PMC8932258

[36] Gabriel AC Bell CN Bowie JV LaVeist TA Thorpe RJ. The Role of social support in moderating the relationship between race and hypertension in a low-income, urban, racially integrated community. J Urban Health. (2020) 97:250–9. 10.1007/s11524-020-00421-1 31997139 PMC7101450

[37] Zhang B Zhang W Sun X Ge J Liu D. Physical comorbidity and health literacy mediate the relationship between social support and depression among patients with hypertension. Front Public Health. (2020) 8:304. 10.3389/fpubh.2020.00304 32850572 PMC7419472

[38] Zhang Q Huang F Zhang L Li S Zhang J. The effect of high blood pressure-health literacy, self-management behavior, self-efficacy and social support on the health-related quality of life of Kazakh hypertension patients in a low-income rural area of China: a structural equation model. BMC Public Health. (2021) 21:1114. 10.1186/s12889-021-11129-5 34112122 PMC8194055

[39] Qiao L Ding S Zhong Z Liu X Lai L Zheng F. Association between social support and medication literacy in Chinese patients with coronary heart disease. Front Cardiovasc Med. (2021) 8:705783. 10.3389/fcvm.2021.705783 34901201 PMC8655157

[40] Sun Z Xu Y. Medical Statistics. People's medical publishing house Co., LTD (2016).

[41] Plaza-Zamora J Legaz I Osuna E Pérez-Cárceles MD. Age and education as factors associated with medication literacy: a community pharmacy perspective. BMC Geriatr. (2020) 20:501. 10.1186/s12877-020-01881-5 33238894 PMC7687724

[42] Zheng F Ding S Zhong Q Pan C Xie J Qin C. Investigation on status of discharged patients' medication literacy after coronary artery stent implantation. Chin Nurs Res. (2015) 29:1732–4. 10.3969/j.issn.10096493.2015.14.024

[43] Wu J Xu J Chen C Wang J Zhao W. Effect of years of education on memory function in patients with hypertension. Hebei Med J. (2012) 34:1345–6. 10.3969/j.issn.1002-7386.2012.09.033

[44] Park JH Floyd JS Park S Kim HC. Combined effect of income and medication adherence on mortality in newly treated hypertension: nationwide study of 16 million person-years. J Am Heart Assoc. (2019) 8:e013148. 10.1161/JAHA.119.013148 31422733 PMC6759906

[45] Huang J. Quantitative Assessment of the Risk Factors for Hypertension Incidence in the Population With Prehypertension in Tianjin. Tianjin: Tianjin medical university (2016).

[46] Wang X Wang X Ma H. Rating Scales for Mental Health. Beijing: Chinese Journal of Mental Health (1999).

[47] Zhang M Liu Y Zhang WY Yang JG Yang WM Zhou J. Exploring perceived challenges of self-management in low-income older people with hypertension: A qualitative study. Int J Nurs Pract. (2022) 28:e13059. 10.1111/ijn.13059 35437909

[48] Rui X. Study of Current Status of Mental Health Literacy and Its Impact on Attitudes Toward Seeking Professional Psychological Help Among Elderly Patients With Hypertension. Zhengzhou: Zhengzhou University (2021).

[49] Ban J Fa T. The correlation between medication literacy and social support in discharged patients after emergency PCI. J Nurses Train. (2020) 35:15–7. 10.16821/j.cnni.hsjx.2020.01.004

[50] Lu L Chen Q. Social support for the elderly in Jiangsu Province. Chin J Gerontol. (2018) 38:5582–4.

