# Supplementary material for: Association between social support and medication literacy in older adults with hypertension
Source: Front Public Health. 2022 Nov 7;10:987526. doi: 10.3389/fpubh.2022.987526 (PMC9677095; doi:10.3389/fpubh.2022.987526)
Supplement: Supplementary file 1 [file Table_1.DOCX]

**APPENDICES:**

**Chinese Medication Literacy Scale for Hypertensive Patients**

**(C-MLSHP)**

1. **Knowledge**

Attached following is a list of statements about hypertension disease, treatment and antihypertensives. Please make a judgment about following statements whether they are correct or not according to your own knowledge, and mark in the corresponding box (true, false and don’t know).

**1.1 Knowledge about hypertension disease and treatment.**

| Items | Statements | True | False | Don’t Know |
| --- | --- | --- | --- | --- |
| K1 | The diagnostic criterion of hypertension is that the BP value≥140/90mmHg in three successive measurements |  |  |  |
| K2 | Hypertension is the leading cause of stroke and coronary heart disease |  |  |  |
| K3 | Hypertension can be induced by weight gain and obesity |  |  |  |
| K4 | Hypertension treatment includes pharmacological therapy and non-pharmacological therapy |  |  |  |
| K5 | Long-term and lifetime antihypertensive therapy is needed for hypertension |  |  |  |

**1.2 knowledge about antihypertensives**

| Items | Statements | Yes | No |
| --- | --- | --- | --- |
| K6 | I know the name of antihypertensives that I have been taking (often take) |  |  |
| K7 | I know the administration method and dosage of antihypertensives that I have been taking (often take) |  |  |
| K8 | I know the side effects and adverse drug reactions of antihypertensives that I have been taking (often take) |  |  |
| K9 | I know the expire date of antihypertensives that I have been taking (often take) |  |  |

1. **Attitude**

Attached following is a list of statements about attitudes to taking antihypertensives, please read each one carefully and choose to tick in one of the boxes (totally disagree to totally agree) which best describes your own opinions about taking antihypertensives.

| Items | Statements | Totally agree | Agree | Not sure | disagree | Totally disagree |
| --- | --- | --- | --- | --- | --- | --- |
| A1 | Patients with hypertension should visit doctors periodically |  |  |  |  |  |
| A2 | Hypertension can have a deleterious influence on health, work and life |  |  |  |  |  |
| A3 | Keep faith that the antihypertensives physician has prescribed could help to control blood pressure and improve health condition |  |  |  |  |  |
| A4 | It is ok to skip or quit taking antihypertensives when I feel good |  |  |  |  |  |
| A5 | Antihypertensives can be quitted once successful or goal blood pressure control is achieved |  |  |  |  |  |
| A6 | I feel that it is too much bother adhering to taking antihypertensives every day |  |  |  |  |  |
| A7 | It’s in way over my head remembering to take antihypertensives every day |  |  |  |  |  |
| A8 | I think it is no big deal to miss a couple of antihypertensive administrations |  |  |  |  |  |

1. **Behavior**

Attached following is a list of questions and statements about some related behaviors of hypertensive patients when they are taking antihypertensives. Please read each question carefully and choose one answer which best describes your opinions or experiences.

**P1** have you ever searched for any information about antihypertensives?

A. Always B. Often C. Sometimes D. Seldom E. Never

**P2** when do you usually search for information about antihypertensive drugs?

1. Prior to and after taking antihypertensive
2. Prior to taking antihypertensives or after physician’s medication prescription
3. After taking antihypertensives
4. After other’s recommendations of a certain kind of antihypertensive
5. Irregular

**P3** I recommend and disseminate antihypertensives with desirable effectiveness to other people (relatives or friends).

A. Always B. Often C. Sometimes D. Seldom E. Never

**P4** how often do you seek advice from medical professionals (physician, pharmacist, and nurse) about information about antihypertensives?

A. Always B. Often C. Sometimes D. Seldom E. Never

**P5** I purchase antihypertensives according to physician’s prescriptions.

A. Always B. Often C. Sometimes D. Seldom E. Never

P6 I self-report adverse drug reactions to medical professionals (physician, pharmacist, and nurse)

A. Always B. Often C. Sometimes D. Seldom E. Never

P7 When you are taking antihypertensives, whether or not they are taken in ways that are in accordance with doctor’s advice or pharmaceutical instructions? please tick in the box of yes or no according to your own experiences.

| Items | Activities | Yes | No |
| --- | --- | --- | --- |
| P7.1 | Dosage of antihypertensives |  |  |
| P7.2 | Time of taking antihypertensives |  |  |
| P7.3 | Shifting the type of antihypertensive drugs |  |  |
| P7.4 | Quitting antihypertensive drugs |  |  |

P8 how often do you have a blood pressure measurement?

1. Every day. B. once every 2~3 days. C. 4~7 days. D. over 7 days

E. irregular

P9 Attached are two statements about matters needing attention when you are doing BP measurement. Please tick in the box of yes or no according to your own experiences.

| Items | Statements | Yes | No |
| --- | --- | --- | --- |
| P9.1 | Whether there is a 20-minute break prior to your every BP measurement |  |  |
| P9.2 | Whether you take a record of BP value every time after you finish the BP measurement |  |  |

1. **Skills**

Attached are two scenario cases with respect to reading and understanding the physician’s prescription and pharmaceutical instruction. Followed with each case, there are several questions about how these prescribed drugs should be administered correctly, please read each case carefully and answer each question by choosing one option which you think is the correct answer according to corresponding cases.

**Case 1:** Tom have been diagnosed as hypertension and he is always feeling headache and dizziness, the latest BP measurement showed 170/110mmHg. The following is a prescription instruction sheet after visiting a physician. Please read the instruction and answer corresponding questions.

| **Data**: 2016-04-10  **Tel:** 0731-88638888 |
| --- |
| **Physician of prescription**: Professor Getz |
| **Metoprolol Succinate Sustained-release Tablets**. 23.75mg. Daily in the morning. (dosage forms and strengths: 47.5mg/tablet)  **Amlodipine Besylate Tablets.** 10mg. Daily in the morning.  **Aspirin Enteric-coated Tablets.** 50mg. Daily before sleeping  **Notes:**  The blood pressure should be measured and recorded after taking antihypertensives.  Come back to the hospital for a check in a month.  Quit smoking, restrict alcohol, have a low-salt diet, and take exercises appropriately |

**S1** According to this prescription sheet, how many times a day should Tom take antihypertensives in total?

Once a day twice a day three times per day four times per day

**S2** what is the date Tom go back to physician for a check the next time?

2016-04-24 2016-5-10 2016-5-22 2016-6-5

**S3** how many pills of metoprolol succinate sustained-release tablets does Tom need?

One tablet. 1/2 tablet. Two tablets. 1/4 tablet

**S4** what measures should be taken in your daily life besides taking antihypertensives as prescribed?

BP measurement. Low-salt diet. Quit smoking. Restricting alcohol. Exercise appropriately. All of the above.

**Case 2: Attached is part of the pharmaceutical instructions of Metoprolol Succinate Sustained-release Tablets Tom has been taking, please read it and answer questions.**

| **Instructions for Metoprolol Succinate Sustained-release Tablets** |
| --- |
| **【Indications】**Hypertension, Angina pectoris, Chronic heart failure with stable left ventricular systolic dysfunction |
| **【Dosage and Administration】**Given orally, once per day, better given in the morning, one tablet can be split apart but not chewed or pulverized when it is given, suggested dose should be taken with at lease half cup of water, dosage should be individualized and should avoid the occurrence of bradycardia. |
| **【Warnings and Precautions】**The possible side effects may be vertigo and fatigue, therefore activities that require people to concentrate such as driving and machine operation should be cautiously considered or avoid. |
| **【Storage】**Keep away from sunlight and sealed in dry place |

**S5** what are the indications of Metoprolol Succinate Sustained-release Tablets?

Hypertension. Angina pectoris. Chronic heart failure with stable symptoms.

All of the above

**S6** Tom is a construction worker driving bulldozer, is there any influence on his work by taking this medicine?

No influence. Have no idea. Cautiously considered

**S7** please tell Tom how to preserve half tablet left along with other medications?

Put them together. Free placed. Preserved as required in the instruction.
